# Cdk5 mediates impaired autophagy by regulating NGF/Sirt1 axis to cause diabetic islet β cell damage

**DOI:** 10.3389/fcell.2025.1613081

**Published:** 2025-10-16

**Authors:** Yuejia Tao, Yipeng Liu, Shijie Hou, Lijun Tang, Kai Wei, Shanshan Zheng, Ying Zhang, Zunsong Wang, Shunyao Liu

**Affiliations:** ^1^ Department of Pathology, The First Affiliated Hospital of Shandong First Medical University & Shandong Provincial Qianfoshan Hospital, Shandong Institute of Nephrology, Shandong Lung Cancer Institute, Jinan, China; ^2^ Department of Pathology, The Fourth People’s Hospital of Jinan, Jinan, China; ^3^ Department of Nephrology, The First Affiliated Hospital of Shandong First Medical University & Shandong Provincial Qianfoshan Hospital, Shandong Institute of Nephrology, Jinan, China; ^4^ Department of Nephrology, Ruijin-Hainan Hospital Shanghai Jiao Tong University School of Medicine (Hainan Boao Research Hospital), Qionghai, China

**Keywords:** Cdk5, diabetes, NGF, Sirt1, autophagy

## Abstract

The number of patients with diabetes is increasing annually, and islet β cell damage plays a central role in the occurrence and development of diabetes. The activation of cyclin-dependent kinase 5 (Cdk5) is involved in the development of diabetes; however, its specific mechanism has not been fully elucidated. This study aimed to investigate the role of Cdk5 in diabetic islet β cell injury. Our results indicate that Cdk5 is upregulated in islet β cells under diabetic conditions, which results in impaired autophagy, and that its inhibition mitites islet β cell injury. In addition, high glucose decreased the levels of nerve growth factor (NGF) and Sirtuin 1 (Sirt1). NGF knockdown was associated with Sirt1 downregulation, while its overexpression upregulated Sirt1, restored autophagy, indicating that NGF positively regulates Sirt1 in islet β cells. Finally, we found that the NGF inhibitor K252a attenuated the protective effect of Lv-Cdk5 shRNA against high glucose-induced islet β cell injury in a mouse model. In conclusion, Cdk5 negatively regulates the NGF/Sirt1 axis, resulting in impaired autophagy of islet β cells under high glucose environments, which lead to islet β cell dysfunction. The Cdk5-NGF/Sirt1 axis may be a new target for the treatment of diabetes.

## 1 Introduction

Diabetes mellitus is considered the most common disease of the 21st century, with the number of diabetes mellitus cases increasing rapidly worldwide. Microvascular and macrovascular complications are the main causes of death in patients with diabetes (Wei et al., 2022). Therefore, preventing and delaying the development of diabetes are important issues in the medical community worldwide. During the occurrence and development of diabetes, the damage of islet β cell function gradually progresses from reversible to irreversible, insulin secretion is reduced, and diabetes and its complications are gradually aggravated (Cerf, 2020).

Cyclin-dependent protein kinases (Cdks) are serine/threonine-specific protein kinases that drive the cell cycle and participate in important cellular processes such as gene transcription and neuronal function through chemical interactions with serine/threonine-specific proteins (Wood and Endicott, 2018). Cdk5, a member of the Cdk family, is crucial for various cellular developmental processes in the brain following binding to p35 or p39, and its disorder is related to the mechanisms of a variety of neurodegenerative diseases (Allnutt et al., 2020). Cdk5 and p35 are also present in islet β cells and participate in the regulation of insulin secretion (Liu S. et al., 2023). However, the mechanism of Cdk5 in diabetic islet β cell damage has not been fully elucidated.

Nerve growth factor (NGF) is a neurotrophic factor that plays an important role in maintaining the growth, differentiation, and other physiological functions of neurones. Studies have found that the increased secretion of NGF protects nerve cells and podocytes from damage caused by high glucose (HG) toxicity (Wu et al., 2020; Cao et al., 2022). In addition, NGF can alleviate the decrease in the number of islet β cells, the average islet area, and the streptozotocin-induced disruption of islet morphology and structure (Ostrovskaya and Ivanov, 2022). Silent information regulator 1 (Sirt1), a substrate of Cdk5 (Bai et al., 2012), is a member of the sirtuin protein family, which has a wide range of biological functions, including maintaining genetic structure, controlling the cell cycle, and regulating metabolism. It is also often used as an anti-inflammatory agent. Sirt1 protects podocyte structure and function against an HG environment (Wang et al., 2025; Lou et al., 2024). Such environments can induce Cdk5 to hyperphosphorylate Sirt1 and lose its protective effect (Bai et al., 2014). Recent studies have revealed that the NGF/Sirt1 axis may be a new therapeutic target for treating cell damage. NGF participates in the protective effect against oxidative damage to hepatocytes by upregulating Sirt1 in human Huh-7 cells and rodent hepatocytes (Tsai et al., 2018). In addition, formononetin protects diabetic rats from hyperglycaemia-induced neuronal damage by controlling hyperglycaemia and upregulating NGF and Sirt1 in nerve tissues (Oza and Kulkarni, 2020). However, whether the NGF/Sirt1 axis exerts the same protective effect on diabetic islet β cells remains unclear.

We previously performed transcriptome sequencing in patients with diabetes and diabetic nephropathy and found that autophagy dysfunction may induce the rapid progression of diabetes to diabetic nephropathy (E et al., 2024). In the present study, we investigated the role of the Cdk5-NGF/Sirt1 axis in modulating autophagy in diabetic islet β cell injury. We found that HG induces increased Cdk5 expression in islet β cells, and Cdk5 negatively regulates the NGF/Sirt1 axis, and this results in the impaired autophagy of islet β cells that lead to islet β cell dysfunction. Our findings may provide a direction for the targeted therapy of diabetes.

## 2 Materials and methods

### 2.1 Antibody

Antibodies against p35/25 (C64B10) was purchased from Cell Signalling Technology (Danvers, MA). Anti-Cdk5 (68514-1-Ig), anti-Beclin1 (66665-1-Ig), anti-p62 (66184-1-Ig), and anti-LC3B (18725-1-AP) antibodies were purchased from Proteintech (Wuhan, China). Anti-β-tubulin (AC008) and LAMP1 (A24804PM) antibodies were obtained from ABclonal (Wuhan, China). Roscovitine was purchased from Dalian Meilun Biotechnology Co. Ltd. K252a (HY-N6732) was obtained from MedChemExpress. NGF small interfering RNA (siRNA) and overexpression plasmids were purchased from Gemma Inc. (PA, USA). Lv-Cdk5 shRNA was purchased from Gemma Inc.

### 2.2 Human pancreas and kidney tissues

We collected paraffin pancreatic tissue sections from 20 patients hospitalised in the First Affiliated Hospital of Shandong First Medical University between August 2023 and August 2024, including patients with (n = 10) and without (n = 10) type 2 diabetes. Pancreatic tissues were obtained from patients undergoing pancreatectomy for pancreatic cancer. The surgical margins were pathologically confirmed as normal pancreatic tissue without other autoimmune diseases, hypertension, or a history of severe infection. The characteristics of the patients are shown in Supplementary Table S1. Written informed consent was obtained from all patients before the collection of pancreatic tissues. This study was approved by the Ethics Committee of the First Affiliated Hospital of Shandong First Medical University (no. 2021-S583).

### 2.3 Animals

Ten 8-week-old male db/db mice (30–35 g) and five male db/m mice (22–26 g) were obtained from Southern Model Animal Co., Ltd. (Nanjing, China). db/db mice were randomly divided into two groups: db/db (n = 5) and db/db + Lv-Cdk5 shRNA group (n = 5). All mice were maintained under a 12 h light/dark cycle, had free access to standard food and water (Wang et al., 2018). To verify the protective effect of Cdk5 inhibition on the pancreas of db/db mice, five 10-week-old db/db + LV-Cdk5 shRNA mice were injected with 40 μL of 1 × 10^6^ infective units of LV-Cdk5 shRNA via the tail vein. The mice in the control and db/m groups were injected with the same volume of normal saline. After 2 weeks of treatment, the anaesthetised mice were tested for blood glucose and were sacrificed, after which the pancreas were harvested for related studies. All experimental procedures were approved by the Animal Care and Use Committee of the First Affiliated Hospital of Shandong First Medical University (no. 2021-S582). All experiments complied with the Guide for the Care and Use of Laboratory Animals.

### 2.4 Intraperitoneal glucose tolerance test (IPGTT)

First, fasting blood glucose was measured after 15 h of fasting. Then the mice were intraperitoneally injected with glucose solution at 2 g/kg, and blood glucose was monitored at 15 min, 30 min, 60 min, 90 min and 120 min, respectively.

### 2.5 Cell culture and transfection

MIN6 cells (mouse islet β cells) were obtained from the Cell Bank of the Chinese Academy of Sciences (Shanghai, China). The cells were incubated in DMEM (Gibco, Carlsbad, CA, USA) supplemented with 10% foetal bovine serum (Gibco) and 1% penicillin and streptomycin (Beyotime, Shanghai, China) in an incubator at 37 °C. The glucose concentrations in the control and HG media were 5 and 25 mM, respectively. The siRNA and overexpression plasmids were transfected using Lipofectamine 3000 (Shanghai Jima Biotechnology Co., Ltd., Shanghai, China) according to the manufacturer’s instructions. Briefly, siRNA was transfected at a final concentration of 100 nmol/L, and plasmid DNA was added to the cultured cells in the six-well plates at a ratio of 1 DNA (µg):2 Lipo3000 (µL). After 6 h, the medium was replaced with normal medium, and Western blotting or immunofluorescence staining was performed after 24 h.

### 2.6 Insulin secretion test

MIN6 cells were incubated overnight in basal Krebs Ringer bicarbonate-HEPES buffer solution (i.e., KRBH) without glucose and were then cultured for 2 h in KRBH containing either normal glucose (5 mM) or HG (25 mM) concentrations. The amount of insulin in the supernatant was measured using a Mouse Insulin Enzyme-Linked Immunosorbent Assay (ELISA) Kit (Elabscience, Wuhan, China).

### 2.7 Western blotting

Western bloting analysis was performed as previously described (Liu S. et al., 2023).

### 2.8 Real-time polymerase chain reaction

The detailed steps of real-time polymerase chain reaction are the same as in our previous article (Liu S. et al., 2023). The primers used were as follows: NGF-F: 5′-TGGAGGTCAATCAACTTGAGAA-3′,NGF-R:5′-CAAACGCAAATGCTTGATCTT-3; Sirt1-F:5′-TGATTGGCACCGATCCTCG-3′,Sirt1-R:5′-CCACAGCGTCATATCATCCAG-3′.

### 2.9 Immunofluorescence staining

The detailed procedure of immunofluorescence staining of pancreatic tissue is the same as in our previous article (Tao et al., 2025). For cellular immunofluorescence staining, the fixed cell slides were placed into a new 12-well plate (or 3 μm paraffin sections were routinely dewaxed) and then permeabilised with 0.3% Triton X-100 at room temperature for 20 min. The following methods of serum sealing, antibody incubation, fluorescence image photography and analysis are the same as the procedure of tissue immunofluorescence staining (Tao et al., 2025).

### 2.10 Immunohistochemical staining

The detailed procedure for immunohistochemical staining was as described in our previous study (Liu S. et al., 2023).

### 2.11 Statistical analysis

GraphPad prism 9.0 software (GraphPad Software, Inc., CA, USA) was used for statistical analysis. Data are expressed as the mean ± standard deviation. *t*-test was used for comparisons between two groups, and one-way analysis of variance was used for multiple-group comparisons. Results were obtained from at least three independent experiments. Statistical significance was set at *P* < 0.05.

## 3 Results

### 3.1 Cdk5 expression was upregulated in islet β cells under high glucose environment

**FIGURE 1:**
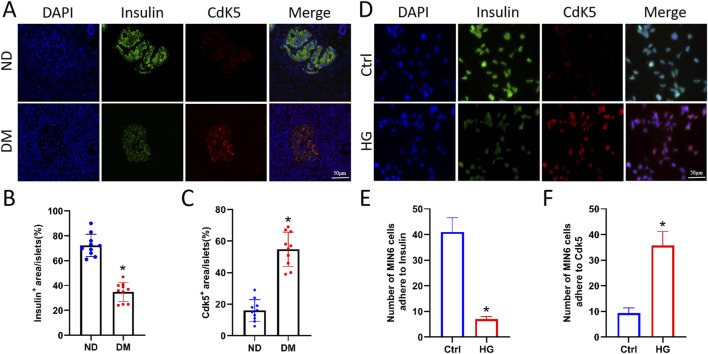
Cdk5 expression was upregulated in islet β cells under high glucose environment. **(A–C)** Immunofluorescence staining of Cdk5 and insulin in human pancreatic tissues. ^*^ *P* < 0.05 vs. ND group. **(D–F)** Cellular immunofluorescence staining was used to detect Cdk5 and insulin expression in Min6 cells (mouse islet β cells). ^*^ *P* < 0.05 vs. Ctrl group.

To verify the expression level of Cdk5 in the islets, pancreatic tissues from patients with clinically diagnosed diabetes were analysed via immunofluorescence staining. Compared with those in the non-diabetic (ND) group, the expression level of Cdk5 was significantly higher while that of insulin was significantly lower in the islets of patients with DM (Figures 1A–C), and Cdk5 colocalised with the islet β cell marker insulin. In addition, Cdk5 and insulin expression in Min6 cells (mouse islet β cells) was detected via cellular immunofluorescence staining, which showed that the two were colocalised and Cdk5 fluorescence was stronger in cells treated with high glucose (Figures 1D–F).

### 3.2 Roscovitine alleviated HG-induced islet β cell injury by inhibiting Cdk5

**FIGURE 2:**
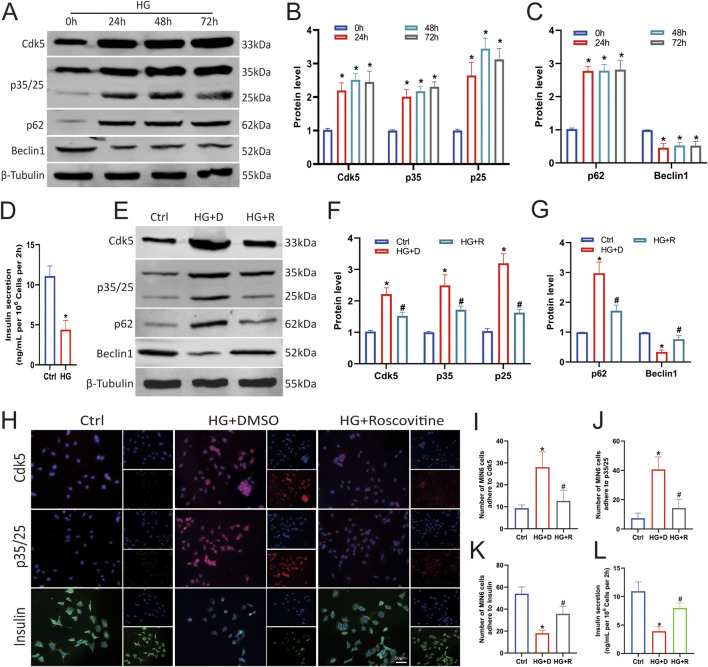
Roscovitine alleviated HG-induced islet β cell injury by inhibiting Cdk5 expression. **(A–C)** Protein expression levels of Cdk5, p35/25, p62, and Beclin1 in Min6 cells. **(D)** Insulin concentration in the culture supernatant of Min6 cells was measured using ELISA. **(E–G)** Protein expression levels of Cdk5, p35/25, p62, and Beclin1 in Min6 cells. **(H–K)** Immunofluorescence staining of Cdk5, p35/25, and insulin in Min6 cells. **(L)** Insulin concentrations in the culture supernatant of Min6 cells were measured using ELISA. ^*^ *P* < 0.05 vs. Ctrl group. ^#^ *P* < 0.05 vs. HG+DMSO group.

To determine the effect of high glucose on Cdk5 expression in pancreatic βcells, Min6 cells were treated with 25 mM glucose for varying durations (0, 24, 48, and 72 h as shown in Figures 2A,B), and it was found that HG stimulated increased Cdk5 and p35/25 expression (Figures 2A,B) and decreased insulin secretion (Figure 2D) in Min6 cells. We have previously conducted transcriptome sequencing of patients with diabetes and diabetic nephropathy and found that autophagy dysfunction may be involved in promoting the rapid progression of diabetes to diabetic nephropathy (E et al., 2024). We clarified the effect of HG on autophagy in Min6 cells by examining the level of autophagy under such conditions. Western blotting showed that HG impaired autophagy (increased p62 and decreased Beclin1 levels) in Min6 cells (Figures 2A,C). These results suggest that HG induces autophagy impairment by promoting Cdk5 expression.

To demonstrate whether Cdk5 is required for islet β cell damage under HG conditions, we inhibited its expression with roscovitine, a Cdk5 inhibitor. Expectedly, intervention with 25 μmol/L roscovitine for 24 h significantly inhibited HG-induced Cdk5 and p35/25 overexpression in Min6 cells (Figures 2E,F,H–J), and restored autophagy (Figures 2E,G). Insulin secretion also was restored (Figures 2H,K,L). Thus, inhibiting the high expression of Cdk5 in an HG environment alleviates the impaired autophagy of islet β cells.

### 3.3 Roscovitine counteracted the HG-induced reduction in NGF and Sirt1 expression in islet β cells

**FIGURE 3:**
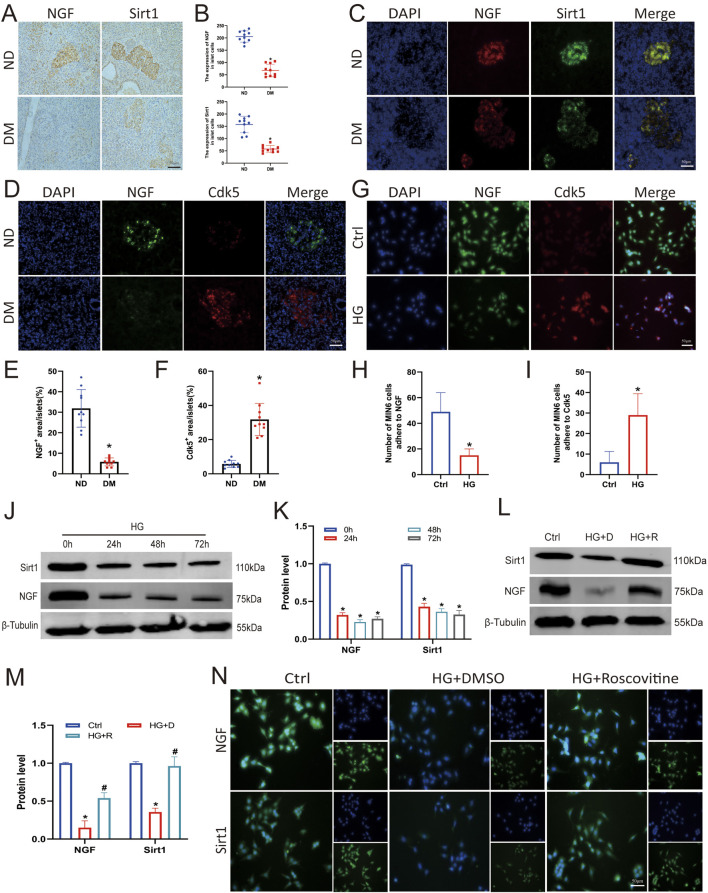
Roscovitine counteracted the HG-induced reduction in NGF and Sirt1 expression in islet β cells. **(A–F)** Immunohistochemical and immunofluorescence staining and statistical analysis of NGF and Sirt1 in human pancreatic tissues. ^*^ *P* < 0.05 vs. ND group. **(G–I)** Immunofluorescence staining was used to detect Sirt1 and NGF expression in Min6 cells. **(J,K)** NGF and Sirt1 protein expression levels in Min6 cells. **(L,M)** NGF and Sirt1 protein expression levels in Min6 cells. **(N)** Immunofluorescence staining of Sirt1 and NGF in Min6 cells. ^*^ *P* < 0.05 vs. Ctrl group. ^#^ *P* < 0.05 vs. HG+DMSO group.

Recent studies have revealed that the NGF/Sirt1 axis may be a new therapeutic target for treating cell damage (Tsai et al., 2018). To verify the expression levels of NGF and Sirt1 in the pancreatic islets, we analysed pancreatic tissues from clinically diagnosed patients with diabetes via immunohistochemical and immunofluorescence staining. The expression levels of NGF and Sirt1 in the islets of patients with DM was significantly lower than those in the islets of ND group, and NGF colocalised with Cdk5 (Figures 3A–F). In addition, cellular immunofluorescence staining showed that NGF and Cdk5 were colocalised in Min6 cells and that the fluorescence of Cdk5 was stronger in the HG-treated group compared with NGF (Figures 3G–I).

To clarify the effect of HG stimulation on NGF and Sirt1 expression, Min6 cells were treated with 25 mM glucose for varying durations. Western blotting showed that HG decreased NGF and Sirt1 expression (Figures 3J,K). To confirm the correlation between Cdk5 knockdown and NGF and Sirt1 expression, we measured NGF and Sirt1 expression levels after roscovitine treatment. Western blotting and immunofluorescence results showed that roscovitine treatment significantly increased NGF and Sirt1 expression levels (Figures 3L–N). These results indicate that roscovitine can rescue the HG-induced reduction in NGF and Sirt1 expression in islet β cells.

### 3.4 Knockdown of NGF attenuated the protective effect of roscovitine on islet β cells

**FIGURE 4:**
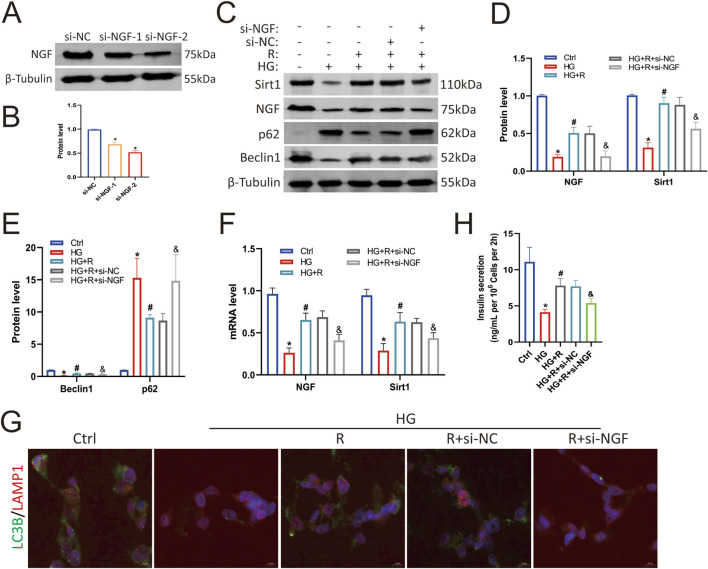
NGF knockdown attenuated the protective effect of roscovitine on islet β cells. **(A,B)** NGF protein expression level in Min6 cells. **(C–E)** Protein expression levels of NGF, Sirt1, p62, and Beclin1 in Min6 cells. **(F)** NGF and Sirt1 mRNA levels were analyzed via Rt-RCR. **(G)** Immunofluorescence staining of LC3B and LAMP1. **(H)** Insulin concentration in the culture supernatant of Min6 cells was measured using ELISA.^*^ *P* < 0.05 vs. Ctrl group. ^#^ *P* < 0.05 vs. HG group. ^&^ *P* < 0.05 vs. HG + roscovitine group.

To determine the protective effect of NGF on islet β cells against an HG environment, Min6 cells were first transfected with NGF siRNA, which resulted in decreased NGF expression (Figures 4A,B). The results of Western blotting showed that roscovitine significantly restored the HG-induced reduction in NGF and Sirt1 expression, restored the expression of autophagy-related proteins p62 and Beclin1, and partially restored insulin secretion (Figures 4C–E,H). However, knockdown of NGF reduced Sirt1 expression in Min6 cells (Figures 4C,D), weakened the protective effect of roscovitine, impaired autophagy (Figures 4C,E), and decreased insulin secretion (Figure 4H). The results of RT-PCR were consistent with the results of Western blotting (Figure 4F). In addition, immunofluorescence staining showed that the co-localization spots of LAMP1 and LC3B were decreased in HG environment, while the co-localization spots of LAMP1 and LC3B were increased after roscovitine treatment; however, the downregulation of NGF attenuated the protective effect of roscovitine (Figure 4G). These results indicate that NGF knockdown reduces Sirt1 expression, impairs autophagy, and attenuates the protective effect of roscovitine on Min6 cells against an HG environment.

### 3.5 Overexpression of NGF alleviated autophagy impairment of islet β cells induced by HG

**FIGURE 5:**
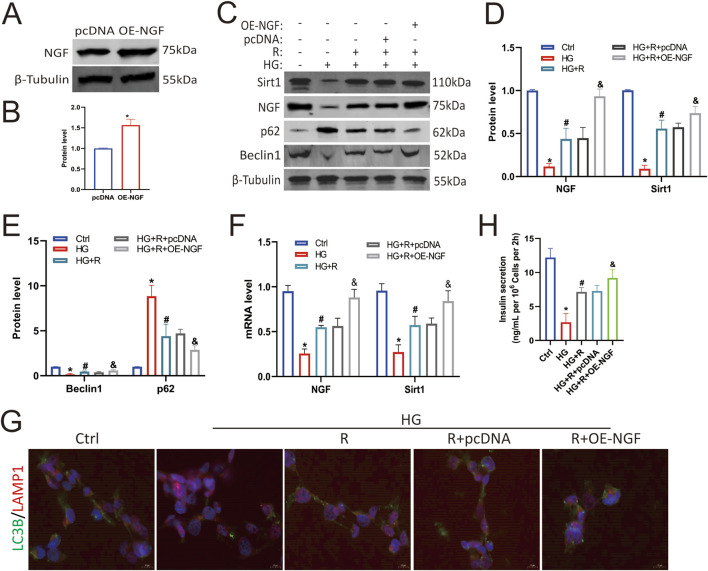
NGF overexpression alleviated autophagy impairment of islet β cells induced by HG. **(A,B)** NGF protein expression levels in Min6 cells. **(C–E)** Protein expression levels of NGF, Sirt1, Beclin1, and p62 in Min6 cells. **(F)** NGF and Sirt1 mRNA levels were analyzed via Rt-RCR. **(G)** Immunofluorescence staining of LC3B and LAMP1. **(H)** Insulin concentration in the culture supernatant of Min6 cells was measured using ELISA. ^*^ *P* < 0.05 vs. Ctrl group. ^#^ *P* < 0.05 vs. HG group. ^&^ *P* < 0.05, vs. HG + roscovitine group.

To further clarify the protective effect of NGF on islet β cells against an HG environment, Min6 cells were transfected with an NGF overexpression plasmid, which resulted in increased NGF protein levels (Figures 5A,B). Western blotting results showed that the overexpression of NGF upregulated Sirt1 (Figures 5C,D), and overexpression of NGF and roscovitine showed a synergistic effect in restoring HG-induced impaired autophagy of Min6 cells (Figures 5C,E). And they increased insulin secretion (Figure 5H). These results suggest that NGF overexpression upregulates Sirt1 and reduces HG-induced autophagy impairment in Min6 cells, demonstrating a synergistic effect with roscovitine. And the results of RT-PCR were consistent with the results of Western blotting (Figure 5F). In addition, immunofluorescence staining showed that the colocalization of LAMP1 and LC3B was decreased in HG environment, which was increased after roscovitine treatment and further increased after upregulation of NGF (Figure 5G). Taken together, these results (Figures 4, 5) suggest that NGF, together with roscovitine, exerts a protective effect by positively regulating Sirt1 expression to alleviate HG-induced impaired autophagy in Min6 cells.

### 3.6 NGF inhibitor attenuated the protective effect of Cdk5 inhibition in diabetic mice

**FIGURE 6:**
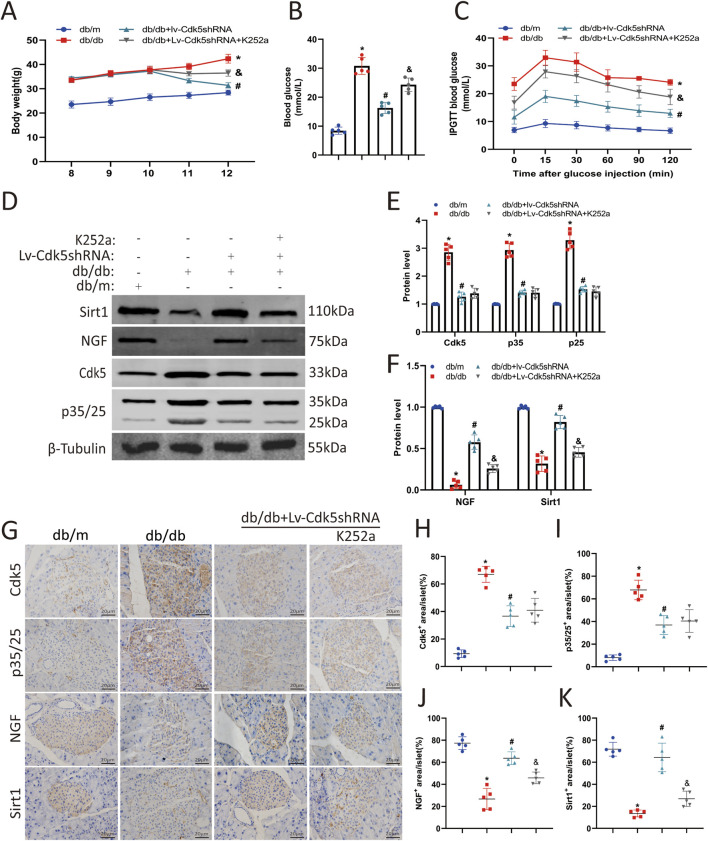
NGF inhibitor attenuated the protective effect of Cdk5 inhibition in diabetic mice. **(A)** Body weight levels of mice in each group. **(B)** Blood glucose levels of mice in each group. **(C)** The IPGTT results of mice in each group. **(D–F)** Protein expression levels of Cdk5, p35/25, NGF, and Sirt1 in mouse pancreas. **(G–K)** Immunohistochemical staining was used to detect the expression of Cdk5, p35/25, NGF, and Sirt1 in pancreatic tissues of mice. ^*^ *P* < 0.05 vs. db/m group. ^#^ *P* < 0.05 vs. db/db group. ^&^ *P* < 0.05 vs. db/db + Lv-Cdk5 shRNA group.

To determine the effects of Cdk5 knockdown and NGF on pancreatic β-cell damage in db/db mice, we treated mice with Lv-Cdk5 shRNA or K252a (NGF inhibitor). We found that Lv-Cdk5 shRNA reduced body weight and blood glucose levels in db/db mice, while K252a attenuated this effect (Figures 6A,B). IPGTT results showed that Lv-Cdk5 shRNA treatment partially restored glucose tolerance in db/db mice, while K252a attenuated the effect of Lv-Cdk5 shRNA (Figure 6C). Western blotting showed that Lv-Cdk5 shRNA treatment significantly decreased Cdk5 and p35/25 expression and increased NGF and Sirt1 levels in the pancreatic tissues of db/db mice, which were attenuated by K252a treatment (Figures 6D–F). Immunohistochemical staining showed the same trend (Figures 6G–K). These results suggest that Lv-Cdk5 shRNA inhibits the high expression of Cdk5 and increases the levels of NGF and Sirt1 in db/db mice and that these effects can be attenuated by an NGF inhibitor.

### 3.7 Inhibition of Cdk5 restored autophagy in islet β cells of db/db mice

**FIGURE 7:**
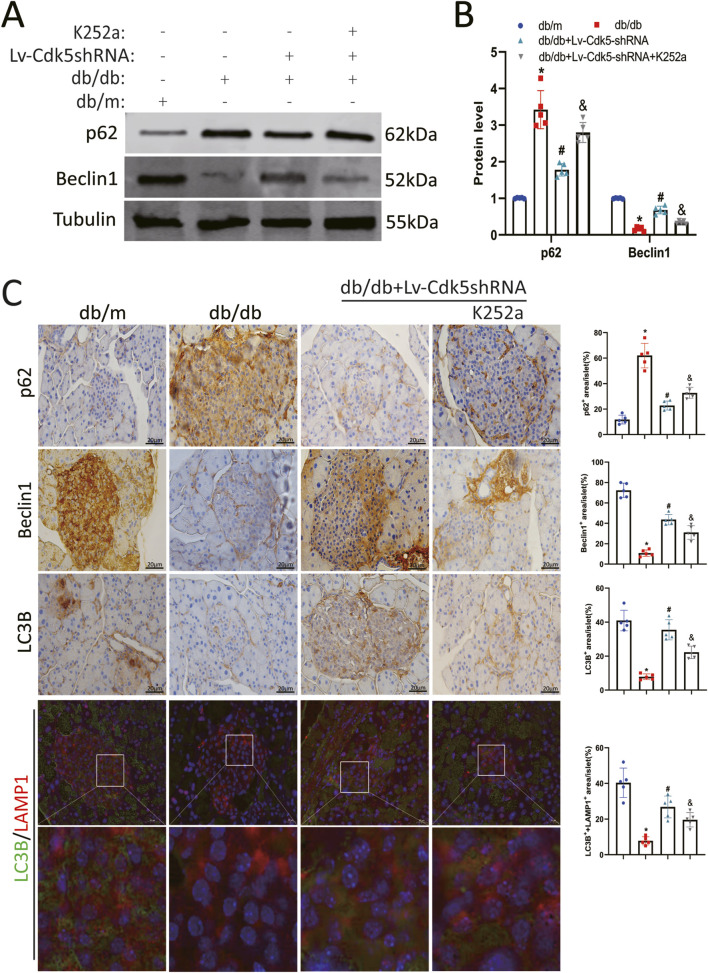
Inhibition of Cdk5 restored autophagy in islet β cells of db/db mice. **(A,B)** Protein expression levels of p62 and Beclin1 in mouse pancreatic tissues. **(C)** Immunohistochemical staining of p62, Beclin1 and LC3B, and immunofluorescence staining of LC3B and LAMP1 in pancreatic tissue of mice. ^*^ *P* < 0.05 vs. db/m group. ^#^ *P* < 0.05 vs. db/db group. ^&^ *P* < 0.05 vs. db/db + Lv-Cdk5 shRNA group.

To investigate the effect of Cdk5 downregulation on autophagy in pancreatic β cells of db/db mice, the expression levels of autophagy-related proteins (p62, Beclin1, and LC3B) in pancreatic tissues of mice treated with Lv-Cdk5 shRNA or K252a were measured. Western blotting and immunohistochemistry staining showed that Lv-Cdk5 shRNA treatment reduced the expression of p62 and restored the levels of Beclin1 and LC3B in db/db mice (Figures 7A–C). However, K252a treatment counteracted these effects, resulting in the upregulation of p62 and downregulation of Beclin1 and LC3B in db/db mice (Figures 7A–C). In addition, immunofluorescence staining showed that the co-localization spots of LAMP1 and LC3B were decreased in db/db mice, while the co-localization spots of LAMP1 and LC3B were increased after Lv-Cdk5 shRNA treatment; however, K252a attenuated the protective effect of Lv-Cdk5 shRNA (Figure 7C). These results suggest that the downregulation of Cdk5 can increase the autophagy of islet β cells in db/db mice, while an NGF inhibitor attenuates these protective effects on islet β cells.

## 4 Discussion

Chronic and progressive damage to islet β cells, particularly through apoptosis, plays a critical role in diabetes development. Studies have found that the islet β cell mass in patients with obesity and diabetes is significantly lower than that in controls with no diabetes, and even patients with impaired fasting glucose have significantly lower values (Matveyenko and Butler, 2008; Lupi and Del Prato, 2008). Therefore, early diagnosis and prevention of diabetes are particularly important (Charles and Leslie, 2021).

Cdk5 is a member of the Cdk family that is not involved in the regulation of the cell cycle but is closely related to neuronal development and activity (Pao and Tsai, 2021). Recent studies have found that Cdk5 is also closely related to non-neuronal functions such as cancer and diabetes and its complications (Nikhil and Shah, 2023; Liu SY. et al., 2023). Cdk5 is expressed in islet β cells and maintains their normal function (Zheng et al., 2010). We have previously found that HG can induce a high expression of Cdk5, leading to inflammation and apoptosis of islet β cells (Liu S. et al., 2023). Cdk5 has also been reported to be an endogenous inhibitor of β cell differentiation, and its inhibition promotes β cell differentiation from ductal progenitor cells, further emphasising its importance in diabetes (Liu et al., 2018).

NGF can protect nerve cells by reducing their apoptosis in HG environments (Wu et al., 2020). In addition, NGF is involved in promoting the maturation process of islet β cells and insulin secretion (Samario-Román et al., 2024). It can also reduce the decrease in the number of islet β cells and the damage of islet morphology and structure caused by streptozotocin (Ostrovskaya and Ivanov, 2022). Sirt1 is a Cdk5 substrate In an HG environment, Sirt1 regulates oxidative stress, reduces inflammation and apoptosis in renal cells, and improves renal interstitial fibrosis in diabetic nephropathy (Ahmed et al., 2019; Dong et al., 2014). In recent years, NGF/Sirt1 has been found to be a potential new therapeutic target for the treatment of cell damage (Tsai et al., 2018). NGF protects diabetic animals from hyperglycaemia-induced neuronal damage by upregulating Sirt1 (Oza and Kulkarni, 2020). In addition, inhibition of Cdk5 overactivation in our diabetic nephropathy model positively regulated the NGF/Sirt1 axis and reduced the HG-induced oxidative stress response and apoptosis in podocytes (Cao et al., 2022). However, the mechanism of the NGF/Sirt1 axis in islet β cell injury has not been fully elucidated.

Defective autophagy is associated with impaired islet β cell function and insulin resistance (Zaidalkilani et al., 2024). We previously performed transcriptome sequencing in patients with diabetes and diabetic nephropathy and found that autophagy dysfunction may promote the rapid progression from diabetes to diabetic nephropathy (E et al., 2024). Canagliflozin protects cardiomyocytes from adriamycin-induced apoptosis by activating SIRT1 to restore autophagic flux (Luo et al., 2025).

In the present study, we examined the expression levels of NGF and Sirt1 in human pancreatic tissue specimens and found that they were lower in islet β cells in patients with diabetes than in those without and inversely correlated with insulin secretion. In addition, we simulated diabetic conditions *in vitro* and found that HG increased Cdk5 expression and decreased NGF and Sirt1 expression in Min6 cells. Inhibition of Cdk5 rescued the HG-induced reduction in NGF and Sirt1 expression, restored autophagy, and restored islet cell function. Next, we used siRNA to knockdown NGF, which reduced the expression of Sirt1, resulting in impaired autophagy of Min6 cells in an HG environment. The use of an NGF overexpression plasmid to increase NGF levels can upregulate Sirt1 and act synergistically with roscovitine to alleviate the damage to the autophagy of Min6 cells in an HG environment. That is, NGF has a positive regulatory effect on Sirt1 expression in Min6 cells, can reduce the HG-induced damage to autophagy of Min6 cells, and has a synergistic protective effect with roscovitine.

We determined the protective effects of Cdk5 knockdown and NGF against pancreatic β-cell injury in db/db mice by treating them with Lv-Cdk5 shRNA or K252a (NGF inhibitor). LV-Cdk5 shRNA inhibited the high expression of Cdk5 in db/db mice, increased the levels of NGF and Sirt1, alleviated the impairment of autophagy in islet β cells, and maintained islet β cell function. However, K252a could weaken the protective effect of Lv-Cdk5 shRNA on islet β cells in db/db mice.

Our study also has some limitations. For example, β-cell damage in db/db mice is mainly caused by defects in the leptin signaling pathway, while human type 2 diabetes is more associated with insulin resistance and inflammation. Furthermore, although MIN6 cells can mimic β-cell function, they have limitations such as low insulin secretion capacity and possible functional decay after long-term passage. In addition, systematic shRNA is suitable for rapid target screening, and systematic knockout mice are more suitable for mechanism verification. It is necessary to further investigate the mechanism in conditioned mice in the future.

In summary, we found that Cdk5 is involved in islet β cell injury under HG conditions. Further mechanistic studies showed that Cdk5 negatively regulates the NGF/Sirt1 axis to induce autophagy impairment of islet β cells, leading to islet β cell dysfunction. The Cdk5-NGF/Sirt1 axis plays an important role in the damage of islet β cells in diabetes and is a new potential target for the treatment of diabetes.

## Data Availability

The raw data supporting the conclusions of this article will be made available by the authors, without undue reservation.
