# Supplementary material for: Cdk5 mediates impaired autophagy by regulating NGF/Sirt1 axis to cause diabetic islet β cell damage
Source: Front Cell Dev Biol. 2025 Oct 16;13:1613081. doi: 10.3389/fcell.2025.1613081 (PMC12573971; doi:10.3389/fcell.2025.1613081)
Supplement: Supplementary file 1 [file Table1.docx]

**Supplements**

**Table 1**

| Characteristics | ND | DM | P value |
| --- | --- | --- | --- |
| Number of patients | 10 | 10 |  |
| Gender (male), n (%) | 4 (40) | 6 (60) | 0.66 |
| Age (years), mean ± SD | 47.7±13.22 | 52.9±12.07 | 0.37 |
| Body Mass Index, mean ± SD | 23.96±3.55 | 24.74±3.15 | 0.61 |
